# Comparative Study of Metallic Iron Production from High-Phosphorus Iron Ores: Carbon-Composite Pellet Direct Reduction–Melting vs. Granular Direct Reduction–Magnetic Separation–Melting

**DOI:** 10.3390/ma19081499

**Published:** 2026-04-09

**Authors:** Bin Wang, Jianjun Gao, Feng Wang, Yue Yu, Yuanhong Qi

**Affiliations:** 1 CISRI Engineering Design Co., Ltd., Beijing 100081, China; wang_bin919@126.com (B.W.);; 2 State Key Laboratory for Advanced Iron and Steel Processes and Products, Central Iron and Steel Research Institute Co., Ltd., Beijing 100081, China; 3 Beijing Steel Research Institute of Hydrometallurgy Technology Co., Ltd., Beijing 100081, China

**Keywords:** high-phosphorus iron ores, direct reduction, metallization, P

## Abstract

High-phosphorus iron ore is an abundant yet refractory resource whose industrial exploitation is severely constrained by its elevated phosphorus content. Guided by the ore’s reduction characteristics, two process routes were designed and compared: carbon-composite pellet direct reduction followed by melting separation (CCP-DR–MS) and direct reduction–magnetic separation–melting separation (DR–MS–MS). Systematic experiments under simulated industrial conditions evaluated the metallization degree, dephosphorization efficiency, and smelting performance while clarifying the key factors that govern the differences in phosphorus removal between the two processes. The results show that both routes raised the ore’s metallization degree to above 95% during direct reduction, but the DR–MS–MS route delivered markedly better dephosphorization, achieving a maximum phosphorus removal rate of 89.20%. Although the slags produced by the two processes showed comparable phosphorus capacities, the difference in overall dephosphorization efficiency stemmed from their distinct reduction mechanisms: in DR–MS–MS, metallic iron is generated mainly via CO-mediated reduction, which suppresses phosphorus reduction, whereas in CCP-DR–MS, phosphorus and iron are simultaneously reduced by carbon. Consequently, DR–MS–MS attains superior phosphorus removal.

## 1. Introduction

With the development of the iron and steel industry, competition has intensified, compelling steel enterprises to adopt low-grade iron ores as substitutes for high-grade ores to reduce production costs, despite the greater smelting challenges associated with low-grade ores [1,2,3]. High-phosphorus iron ores are abundantly available globally, but their utilization remains limited due to their classification as complex refractory ores. These ores exhibit intricate intergrowth relationships between iron minerals and gangue minerals, coupled with high phosphorus content [4,5,6].

Current utilization methods for high-phosphorus iron ores primarily include physical beneficiation, leaching, roasting–magnetic separation, and direct reduction [7,8,9,10,11,12,13,14,15,16,17,18,19]. Recent studies have demonstrated that pyrometallurgical processing of high-phosphorus ores offers advantages such as large-scale processing capacity, strong raw material adaptability, low pollution, and effective dephosphorization [20,21]. To achieve efficient iron recovery and phosphorus removal, the addition of dephosphorization agents is typically required during pyrometallurgical treatment [22,23,24]. Commonly used agents include CaCO_3_, Ca(OH)_2_, Na_2_CO_3_, and Na_2_SO_4_ [11]. Xu et al. [25] found that the addition of Na_2_CO_3_ suppressed the incorporation of phosphorus into metallic iron while reacting with phosphorus in the raw ore to form soluble Na_3_PO_4_. Li et al. [26] proposed that sodium salts not only enhanced the reduction of high-phosphorus ores but also reacted with apatite to form structurally stable CaNaPO_4_. Rao et al. [27] demonstrated that a 20% Na_2_SO_4_ addition was required to convert apatite in a high-phosphorus ore from Hunan Province into stable CaNaPO_4_. Meanwhile, Luo [28] and Zhao [21] et al. discovered that CaCO_3_ and Ca(OH)_2_ additions also facilitated the reduction of high-phosphorus ores while inhibiting the reduction of apatite.

This industry-oriented study used FactSage thermodynamic calculations to systematically examine the processing of high-phosphorus iron ore and to determine the conditions required for phosphorus reduction. Two alternative process routes were then developed and compared—CCP-DR–MS and DR–MS–MS. The experimental matrix varied the Ca(OH)_2_ addition level to clarify how slag basicity, temperature, and holding time affect direct-reduction efficiency. The dephosphorization performance of CCP-DR–MS and DR–MS–MS on the metallized products was subsequently evaluated, and the fundamental causes of the performance differences between the two routes were identified. Finally, a comprehensive test program established the optimum industrial flowsheet and operating parameters.

## 2. Experimental

### 2.1. Raw Materials

**Figure 1:**
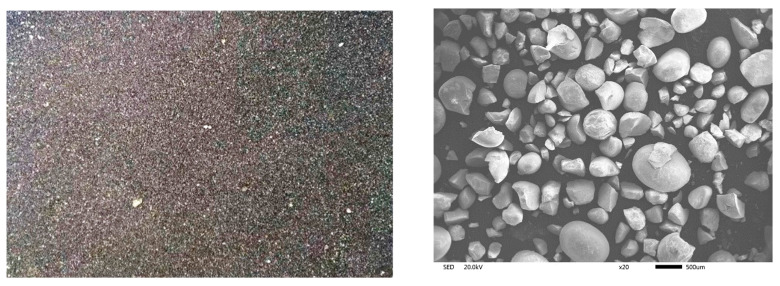
Morphology of washed high-phosphorus ore.

The raw material used in this experiment was a high-phosphorus iron ore provided by a collaborating company. This ore is a placer deposit intermingled with significant amounts of clay. After undergoing water washing and filtration, a sand-like concentrate was obtained (Figure 1). The chemical composition of the washed high-phosphorus ore is presented in Table 1.

**Figure 2:**
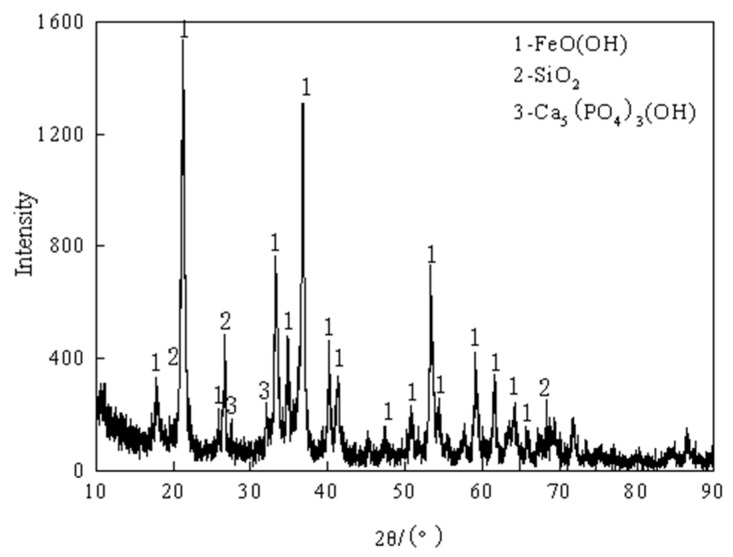
XRD analysis results of high-phosphorus iron ore.

The mineral phase analysis results of the high-phosphorus ore are presented in Figure 2. The findings indicate that iron primarily exists as goethite (FeO(OH)), silicon is predominantly in the form of SiO_2_, and phosphorus is mainly present as apatite (Ca_5_(PO_4_)_3_(OH)). Goethite (FeO(OH)) undergoes thermal decomposition at elevated temperatures according to the following reaction:

2FeO(OH) → Fe_2_O_3_ + H_2_O (1)

This decomposition reaction accounts for the high loss on ignition (LOI) observed in the high-phosphorus ore.

**Figure 3:**
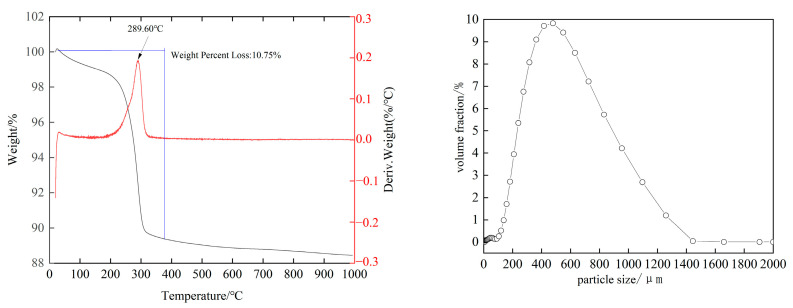
Thermogravimetric and particle size analysis results of high-phosphorus ore.

Figure 3 presents the combined thermogravimetric analysis (TGA) and particle size distribution (PSD) results of the high-phosphorus ore under inert atmosphere conditions. The analysis revealed a strong endothermic peak at approximately 290 °C, accompanied by a mass loss of 10.75%. This phenomenon is primarily attributed to the dehydration of goethite (FeO(OH)) present in the ore. The ore particles were predominantly distributed in the range of 300–1000 μm. The median particle size (d50) was determined to be 471 μm. The size distribution analysis further indicated that 90% of the particles were smaller than 909 μm, while 10% of the particles measured less than 223 μm.

### 2.2. Theoretical Basis of Experiments

**Figure 4:**
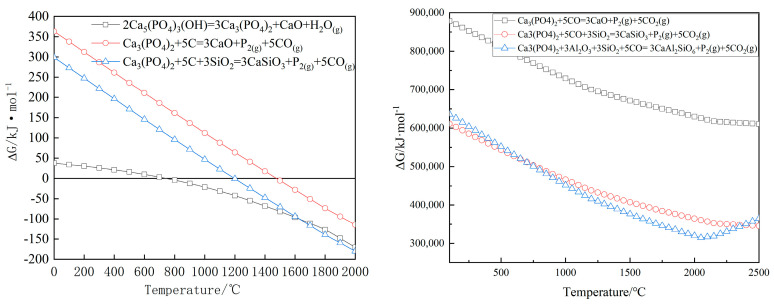
ΔG for C and CO reduction of calcium phosphate.

Phase analysis of high-phosphorus iron ore revealed that phosphorus primarily exists in the form of apatite (Ca_5_(PO_4_)_3_(OH)). Given the complex composition of high-phosphorus ore, SiO_2_ and Al_2_O_3_ inevitably participate in the reduction of apatite during the reduction process. Reactions (2)–(8) represent potential reduction pathways for apatite, with their corresponding ΔG as functions of temperature presented in Figure 4. All Gibbs free energy changes (ΔG) reported in this study are calculated per mole of reaction as written, based on the stoichiometric coefficients in the corresponding balanced chemical equation.

2Ca_5_(PO_4_)_3_(OH) = 3Ca_3_(PO_4_)_2_ + CaO + H_2_O(g) (2)

Ca_3_(PO_4_)_2_ + 5C = 3CaO + P_2_(g) + 5CO(g) (3)

Ca_3_(PO_4_)_2_ + 5C + 3SiO_2_ = 3CaSiO_3_ + P_2_(g) + 5CO(g) (4)

Ca_3_(PO_4_)_2_ + 3Al_2_O_3_ + 3SiO_2_ + 5C = 3CaAl_2_SiO_6_ + P_2_(g) + 5CO(g) (5)

Ca_3_(PO_4_)_2_ + 5CO(g) = 3CaO + P_2_(g) + 5CO_2_(g) (6)

Ca_3_(PO_4_)_2_ + 5CO(g) + 3SiO_2_ = 3CaSiO_3_ + P_2_(g) + 5CO_2_(g) (7)

Ca_3_(PO_4_)_2_ + 3Al_2_O_3_ + 3SiO_2_ + 5CO(g) = 3CaAl_2_SiO_6_ + P_2_(g) + 5CO_2_(g) (8)

The results indicate that apatite begins to decompose at approximately 700 °C, while pure Ca_3_(PO_4_)_2_ can only be reduced by carbon at around 1500 °C. However, the presence of SiO_2_ in the raw ore promotes the reduction of Ca_3_(PO_4_)_2_. Under SiO_2_-bearing conditions, Ca_3_(PO_4_)_2_ can be reduced at approximately 1200 °C. In practical reduction processes, the partial pressure of phosphorus vapor generated is relatively low, leading to an even lower reduction temperature for Ca_3_(PO_4_)_2_. The ΔG values for apatite reduction by CO indicate that CO cannot reduce apatite at lower temperatures.

**Figure 5:**
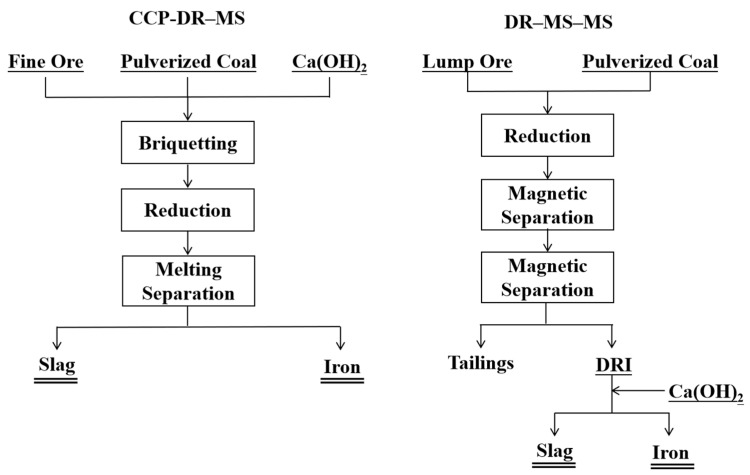
Process flow diagrams.

Based on thermodynamic calculations, two processing routes were designed: the carbon-composite pellet direct-reduction–melting separation (CCP-DR–MS) route and the direct-reduction–magnetic-separation–melting separation (DR–MS–MS) route, as illustrated in Figure 5.

### 2.3. Experimental Methodology

In the direct reduction–melting separation process of carbon-composite pellets, high-phosphorus iron ore was first mixed with pulverized coal and fluxing agents (CaO/Na_2_CO_3_) for pelletization, followed by high-temperature direct reduction and subsequent melting separation to achieve slag–iron separation. The experimental study—conducted under simulated industrial conditions with 60 min reduction heating and 120 min isothermal holding—systematically investigated the effects of flux dosage and melting temperature on metallization efficiency and phosphorus content in iron products. Based on comprehensive cost–benefit analysis, calcium hydroxide (Ca(OH)_2_, ≥99% purity) was selected as the optimal flux, with detailed experimental parameters presented in Table 2.

To investigate the effects of direct reduction temperature and final slag basicity on metallization rate and phosphorus content in the direct reduction–magnetic separation–melting separation process, the high-phosphorus raw ore was first uniformly mixed with pulverized coal, then directly reduced at relatively low temperatures. The reduced material underwent magnetic separation to remove excess coal powder. Finally, samples with better metallization rates were selected for high-temperature melting separation with Ca(OH)_2_ addition. The experimental design is shown in Table 3.

## 3. Results and Discussion

### 3.1. Direct Reduction of the CCP-DR–MS Process

**Figure 6:**
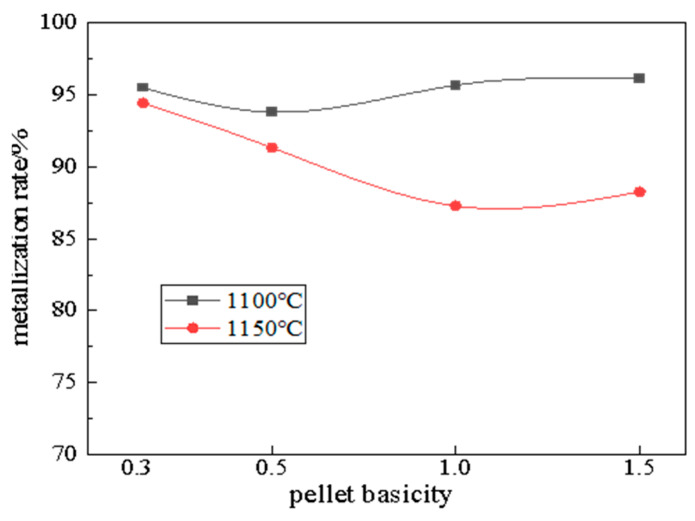
Effect of basicity on pellet metallization rate.

Figure 6 presents the influence of slag basicity on the metallization rate of carbon-composite pellets. The results indicate that the addition of Ca(OH)_2_ had a minimal effect on the metallization rate, with consistently high metallization achieved across all tested basicity conditions. Specifically, pellets reduced at 1100 °C achieved about 95.0% metallization under natural basicity, while Ca(OH)_2_-adjusted basicities of 0.5 and 1.0 yielded 93.80% and 95.67%, respectively, confirming the ore’s inherent reducibility. However, increasing the temperature to 1150 °C generally led to a decrease in the metallization rate. This is because, in the pre-reduction experiments on carbon-bearing pellets, raising the pre-reduction temperature significantly accelerates the reduction reaction rate. Consequently, the pellets complete the reduction process more rapidly at 1150 °C than at 1100 °C. As the reduction time extends, the reaction rate gradually decreases, and the rate of CO generation slows accordingly. Since the pre-reduction process for carbon-bearing pellets does not require an external protective atmosphere, the environment surrounding the pellets gradually becomes weakly oxidizing under these conditions. Based on the above analysis, it can be inferred that the decrease in the metallization rate is attributable to secondary oxidation. These findings underscore that while basicity modification is unnecessary for enhancing metallization, temperature control below 1150 °C is critical to mitigate unintended oxidative losses.

**Figure 7:**
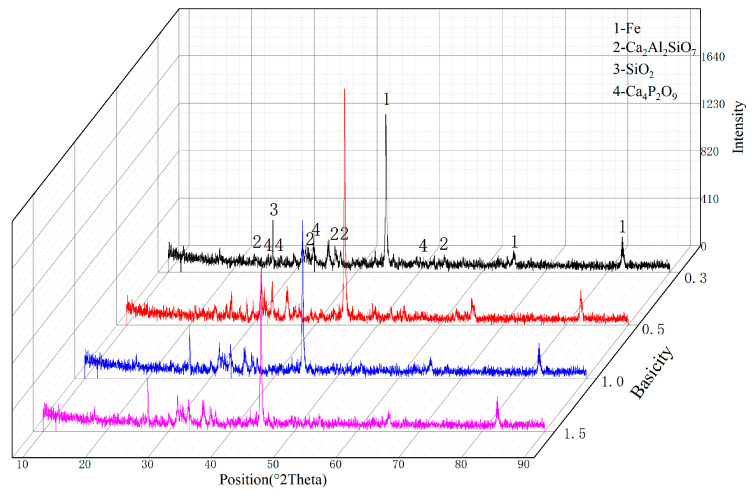
Phase composition of reduced materials.

The phase composition of the reduced material at 1150 °C is shown in Figure 7. The primary phases detected were metallic iron (Fe), gangue compounds (Ca_2_Al_2_SiO_7_), SiO_2_, and calcium phosphate (Ca_4_P_2_O_9_), confirming that basicity had negligible effects on pellet metallization. The formation of Ca_2_Al_2_SiO_7_ indicates that at high temperatures, SiO_2_ and Al_2_O_3_ in the raw ore promoted the reduction of calcium phosphate. However, the complete reduction of calcium phosphate is undesirable in direct reduction, as liberated phosphorus would combine with metallic iron, resulting in high phosphorus content in the final hot metal during melting separation. Notably, Ca_4_P_2_O_9_ was detected under all four basicity conditions, suggesting that not all phosphorus was reduced, with a portion remaining fixed in calcium phosphate. These collective findings demonstrate that the high-phosphorus ore attains excellent reducibility (about 95% metallization rate) within the 1100–1150 °C temperature range without requiring CaO flux addition.

### 3.2. Smelting Separation of the CCP-DR–MS Process

**Figure 8:**
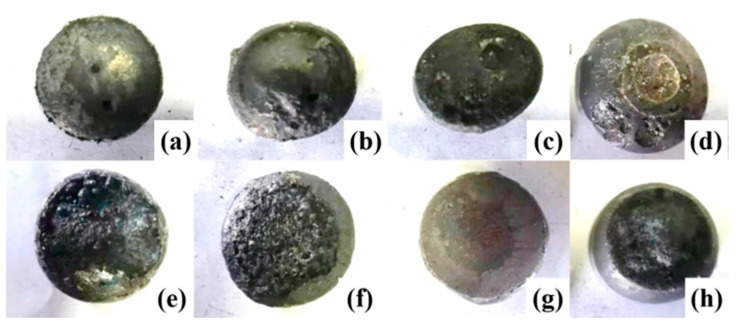
Macroscopic morphology of carbon-composite pellets after melting separation. (**a**) 1400 °C Basicity 0.3, (**b**) 1400 °C Basicity 0.5, (**c**) 1400 °C Basicity 1.0, (**d**) 1400 °C Basicity 1.5, (**e**) 1450 °C Basicity 0.3, (**f**) 1450 °C Basicity 0.5, (**g**) 1450 °C Basicity 1.0, (**h**) 1450 °C Basicity 1.5.

Figure 8 presents the macroscopic morphology of pellets after melting separation, revealing that effective slag–iron separation was achieved at both 1400 °C and 1450 °C across all tested basicity levels, with higher basicity resulting in smoother iron surfaces due to improved slag coverage. Under natural basicity conditions, the metallic iron aggregated into regular spherical shapes at both temperatures but exhibited severe surface oxidation with minor slag adherence, primarily attributed to insufficient slag volume for complete iron encapsulation, leaving the metal vulnerable to atmospheric oxidation. As the basicity increased, the greater slag formation provided protective encapsulation of the iron, significantly reducing surface oxidation and yielding cleaner metal surfaces. This behavior demonstrates the critical role of basicity-controlled slag volume in preventing iron reoxidation during high-temperature processing, where adequate slag fluidity and thickness are essential for maintaining metal quality.

**Figure 9:**
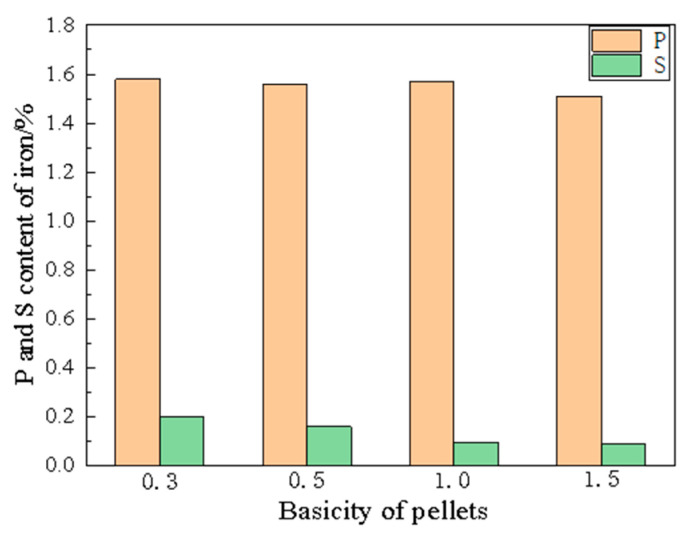
P and S content in metallic iron obtained after melting separation of pellets with different basicity at 1450 °C.

Figure 9 presents the P and S content in the molten iron after melting separation. The results show that the P content remained consistently high at approximately 1.6% across all basicity conditions, indicating the near-complete reduction and transfer of P from the ore into the metal phase. In contrast, the S content exhibited a clear decreasing trend with increasing basicity, demonstrating that the added Ca(OH)_2_ effectively fixed S into the slag phase as CaS. At a basicity of 1.0, the S content was reduced below 0.08%, meeting standard requirements for steelmaking pig iron. However, the persistently high P content (around 1.6%) far exceeds acceptable levels for steel production. These findings clearly demonstrate that conventional direct reduction–melting separation of carbon-containing pellets cannot produce qualified molten iron from high-phosphorus ores.

### 3.3. Direct Reduction of the DR–MS–MS Process

**Figure 10:**
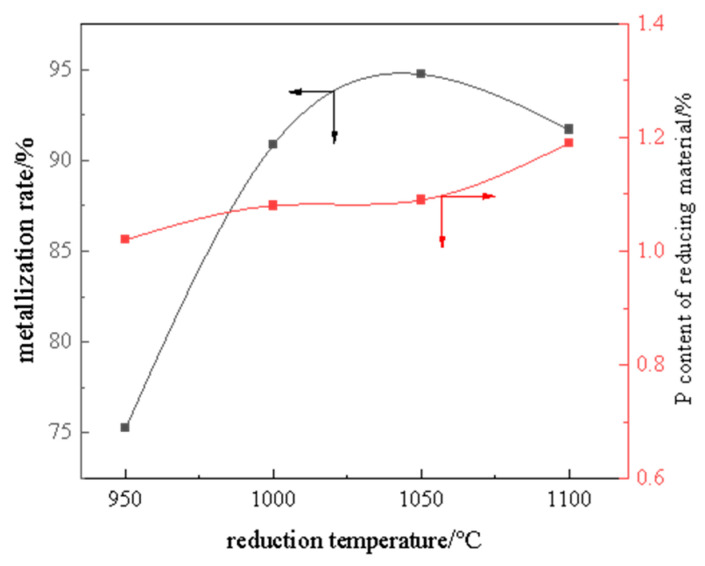
Metallization rate of raw ore reduction and phosphorus content after magnetic separation at different temperatures.

Figure 10 presents the metallization rate of high-phosphorus iron ore and the phosphorus content in reduced materials after magnetic separation at different reduction temperatures. The results show that the metallization rate increases progressively with rising reduction temperature, exceeding 90% when the temperature surpasses 1000 °C. Meanwhile, the phosphorus content in the reduced materials exhibits a gradual but limited increase with temperature, maintaining within the range of 1.0–1.2 wt%.

**Figure 11:**
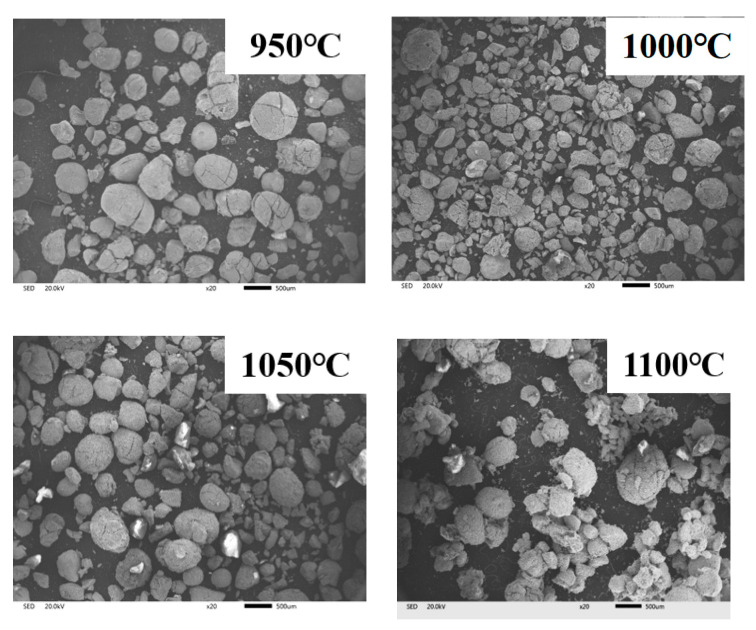
SEM analysis of reduced materials.

**Figure 12:**
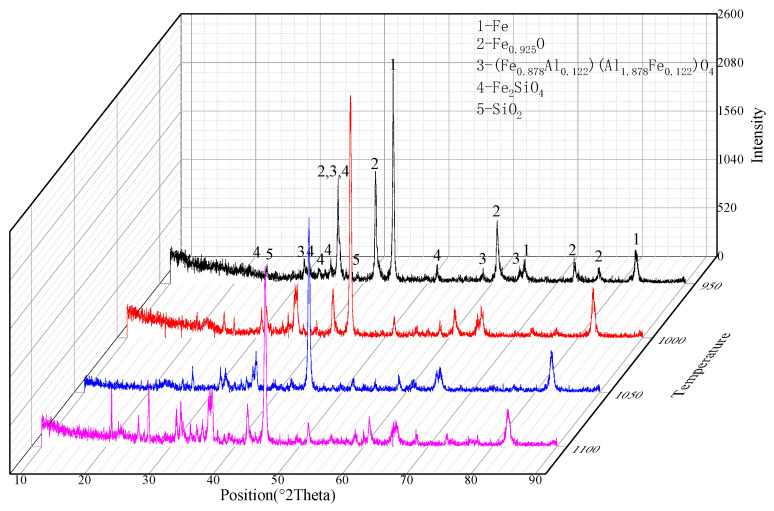
XRD analysis of reduced material.

Figure 11 and Figure 12 present the SEM-EDS and XRD analysis results of reduced materials at different reduction temperatures. At 950 °C, the reduced powder maintained its original particle morphology without agglomeration, though surface cracks were observed, indicating gas evolution from particle interiors that generated pores and cracks. The primary phases identified were Fe, FeO, FeSiO_4_, and SiO_2_, with a metallization rate of approximately 75%. When the temperature increased to 1000–1050 °C, the metallization rate exceeded 90%, demonstrating that elevated temperatures accelerated coal powder gasification and consequently enhanced iron oxide reduction. Although the phase composition showed minimal changes, the markedly intensified diffraction peaks of metallic iron confirmed improved reduction efficiency. SEM-EDS analysis revealed persistent Ca and P signals within iron whiskers, evidencing the tight encapsulation of apatite in the metallic iron matrix that hindered effective separation. While 1100 °C reduction achieved over 90% metallization, partial surface melting and interparticle bonding occurred, suggesting that optimal processing should employ the lowest possible temperature while maintaining high metallization rates for this high-phosphorus ore.

### 3.4. Smelting Separation of the DR–MS–MS Process

**Figure 13:**
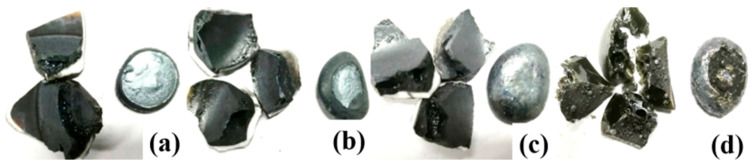
Melting separation results of 1000 °C-reduced charges with different basicities. Technical Description. (**a**) Basicity 0.3, (**b**) Basicity 1.0, (**c**) Basicity 1.6, (**d**) Basicity 2.0.

In industrial production processes, the particle melting and agglomeration of mineral powders frequently lead to a ring formation in rotary kilns or other direct reduction equipment, disrupting continuous operation, reducing production efficiency and equipment service life, and increasing costs. To prevent this phenomenon, the optimal reduction temperature was established at 1000 °C, where the metallization rate exceeds 90% while maintaining a sufficient temperature margin below the particle agglomeration threshold. This temperature selection significantly expands the acceptable operational temperature range in industrial settings and reduces process control requirements. Figure 13 presents the smelting behavior and slag–iron separation results for metallized furnace charge (reduced at 1000 °C) with CaO additions, yielding final slag basicities of 0.3 (natural basicity), 1.0, 1.6, and 2.0 under constant conditions of 1550 °C melting temperature and 40 min holding time. The results demonstrate successful slag–iron separation across all basicity levels, with metallic iron aggregating into smooth iron blocks and slag forming dense, consolidated layers.

**Figure 14:**
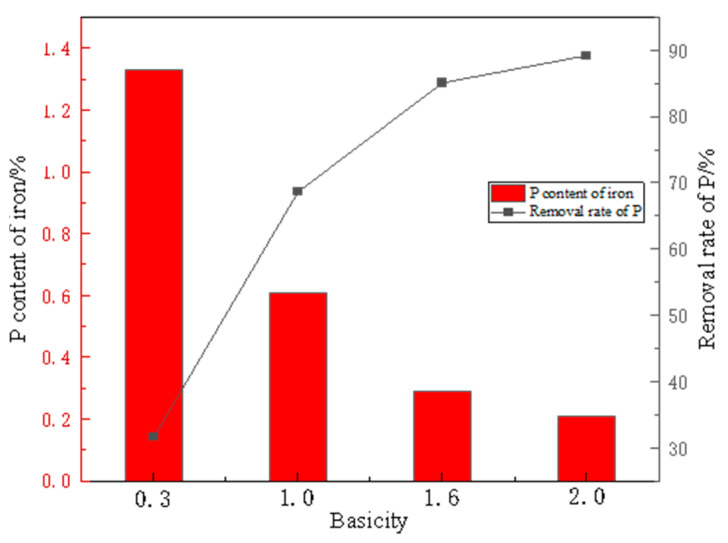
P content in iron nuggets after smelting reduction at different slag basicities.

Figure 14 shows the P content in iron nuggets after smelting reduction at different slag basicities. The P content varied significantly with basicity, decreasing markedly as basicity increased. Without Ca(OH)_2_ addition, the iron nugget contained 1.33% P—0.3% lower than direct reduction-smelting of high-phosphorus pellets but still unacceptably high, indicating poor P removal. When Ca(OH)_2_ was added to achieve a slag basicity of 1.0, the phosphorus content in the iron product decreased to 0.61%, corresponding to approximately 50% phosphorus removal. Further enhancement was observed using CaO as the fluxing agent—at a basicity of 1.6, the phosphorus content dropped to 0.29%, and when the basicity was increased to 2.0, the phosphorus content reached its lowest level of 0.21%. This improvement stems from suppressed phosphate reduction due to low temperature and lack of CaO, preventing P transfer to metallic iron, combined with magnetic separation that removed both coal fines and gangue (directly eliminating some P while inhibiting P reduction during smelting). Although the direct reduction–magnetic separation–smelting process removed >85% P (reducing content to 0.21%), the residual P still exceeds steelmaking requirements, necessitating further deep dephosphorization.

### 3.5. Reasons for Differences in Dephosphorization Efficiency

The experimental results demonstrate that both processes can achieve high metallization rates, yet they exhibit markedly different efficiencies in phosphorus removal. This section examines the disparity in dephosphorization performance between the two processes. As established in metallurgical practice, reducing phosphorus content in metallic iron primarily relies on either preventing phosphorus incorporation into iron or eliminating phosphorus from molten iron through slag reactions. The analysis begins by evaluating the dephosphorization capacity of the slags generated by each process.

During the smelting reduction process, elements detrimental to steel properties such as S and P are oxidized and transferred into the slag. When $P_{O_{2}}$ in the system is below 10^−13^ Pa, phosphorus predominantly exists as P^3-^ ions, whereas when $P_{O_{2}}$ exceeds 10^−12^ Pa, it mainly takes the form of ${PO}_{4}^{3-}$ ions [29]. The relevant chemical reactions can be expressed as follows:

(9) $$\frac{1}{2}P_{2}\left(g\right)+\frac{3}{2}\left(O^{2-}\right)=\left(P^{3-}\right)+\frac{3}{4}O_{2}$$

(10) $$\frac{1}{2}P_{2}\left(g\right)+\frac{3}{2}\left(O^{2-}\right)+\frac{5}{4}O_{2}=({PO}_{4}^{3-})$$

According to the ionic theory, the phosphorus capacity of slag can be expressed by the following formula:

(11) $$C_{{PO}_{4}^{3-}}=\frac{\omega ({PO}_{4}^{3-})}{f_{P}\omega [P]a_{\left[O\right]}^{5/2}}=K\frac{a_{\left[O\right]}^{3/2}}{\Upsilon_{{PO}_{4}^{3-}}}$$

The phosphorus capacity of slags produced by both processes cannot be determined directly from this formula, as the activity coefficients of phosphate ions and oxygen ions in the respective slag systems remain unquantifiable.

The phosphorus capacity of slag can be calculated using the empirical formula derived by Young [30] from experimental data, which is expressed as follows:

(12) $$lgC_{{PO}_{4}^{3-}}=-18.184+35.84\Lambda -23.35\Lambda^{2}+\frac{22930\Lambda }{T}-0.06257\omega \left(FeO\right)-0.004256\omega \left(MnO\right)+0.259\;[\omega P_{2}O_{5}{]}^{0.3}$$

Λ represents the optical basicity of the slag, while ω (FeO), ω (MnO), and ω (P_2_O_5_) denote the mass percentages of FeO, MnO, and P_2_O_5_ in the slag, respectively. For multicomponent slags consisting of various compounds and oxides, the basicity correlates with the activity of O^2−^ ions, which is quantitatively expressed through the optical basicity—a parameter that characterizes the overall electron-donating capacity of all constituent oxides and compounds in the molten slag system. The mathematical expression is given below:

(13) $$\Lambda =\sum_{B=1}^{n}x_{B}\Lambda_{B}$$

where *Λ_B_* represents the optical basicity of the oxide, and *x_B_* denotes the molar fraction of cations in the oxide.

(14) $$x_{B}=n_{O}x_{B}^{\prime }/\sum n_{O}x_{B}^{\prime }$$

where $x_{B}^{\prime }$ represents the molar quantity of the oxide, and $n_{O}$ denotes the number of oxygen atoms in the oxide molecule.

The optical basicity values of common components in slag are presented in Table 4.

**Figure 15:**
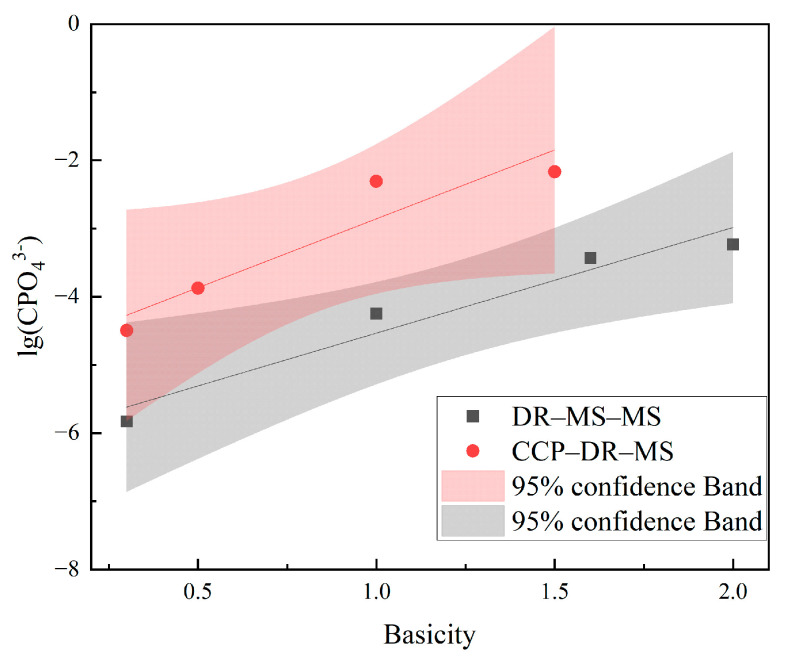
Calculated phosphorus capacity of slags generated by the two processes.

Figure 15 presents the calculated phosphorus capacities of slags produced by the two processes. The results demonstrate that both slags exhibit relatively low phosphorus capacities, with the CCP-DR–MS slag showing marginally better dephosphorization performance than the DR–MS–MS slag. When correlated with the phosphorus content in the resultant iron products, it becomes evident that the dephosphorization effects of both slags have minimal impact on phosphorus removal from the molten iron. These findings conclusively indicate that for iron production using high-phosphorus ores, the primary focus for phosphorus control should be on preventing phosphorus transfer into the molten iron phase rather than relying on slag-mediated removal.

**Figure 16:**
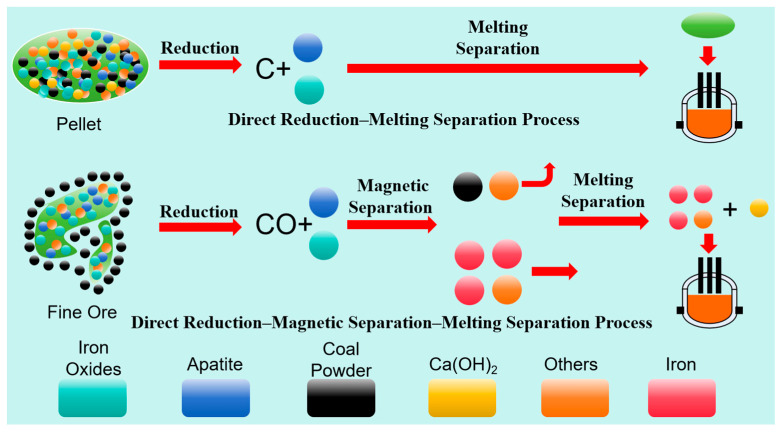
Comparative analysis of key process steps.

Figure 16 reveals the underlying mechanism for this difference. In the carbon-containing pellet direct reduction–melting separation process, the elevated temperature provides favorable thermodynamic conditions for reduction, while the intimate mixing of pulverized coal and ore fines enhances reduction kinetics. Consequently, Fe–P alloy formation occurs due to the simultaneous reduction of iron and phosphorus. In contrast, the direct reduction–magnetic separation–melting separation process exhibits limited contact between coal and raw ore, causing CO to dominate as the reducing agent. Since CO-driven apatite reduction requires higher temperatures, phosphorus remains predominantly as Ca_3_(PO_4_)_2_ during direct reduction, effectively preventing Fe–P alloy generation. Although the complex mineralogical associations in high-phosphorus ore prevent the complete removal of gangue and phosphorus during post-reduction magnetic separation, nearly all residual coal is eliminated. This critically suppresses phosphorus reduction in subsequent melting stages.

The DR–MS–MS process system exhibits a significant decrease in phosphorus content within the metallic phase upon CaO addition, as revealed by experimental investigations. This behavior stems from competitive high-temperature reaction mechanisms wherein SiO_2_ lowers the reduction temperature of Ca_3_(PO_4_)_2_ while, simultaneously, the introduced CaO preferentially reacts with SiO_2_ to form thermodynamically stable Ca_2_SiO_4_ phases. This dual effect not only consumes available SiO_2_ but also enhances the stability of phosphorus compounds, thereby preferentially retaining phosphorus within the slag phase rather than permitting its migration into the metallic phase.

## 4. Conclusions

1. In high-phosphorus iron ore, iron primarily exists in goethite (FeO(OH)) form, while phosphorus is predominantly present as apatite. Thermal analysis reveals that goethite (FeO(OH)) in this ore undergoes decomposition at approximately 290 °C, exhibiting a mass loss rate of 10.75%. Particle size distribution measurements indicate an average ore diameter of 471 μm (approximately 35 mesh), characterizing this as a typical sandy-type high-phosphorus ore with relatively coarse granulometry.
2. The direct reduction of carbon-composite pellets (26% coal addition, 180 min, 1100–1150 °C) achieved ∼95% metallization with facile slag–iron separation. However, phosphorus was synchronously reduced with iron during the process, and Ca(OH)_2_ addition during smelting showed negligible effects on phosphorus removal. Consequently, the resulting iron contained about 1.6% phosphorus—exceeding steelmaking specifications—rendering this route unsuitable for producing a qualified iron product.
3. The granular ore direct reduction process (30% coal, 1050 °C, 120 min) attained 94.72% metallization, 82.37% iron grade, and 1.05% residual phosphorus. Crucially, phosphorus remained unreduced during CO-dominated reduction, while magnetic separation eliminated coal residues and partial gangue. Subsequent smelting with Ca(OH)_2_ (basicity 2.0, 1550 °C, 40 min) yielded iron with only 0.21% phosphorus (>85% removal). With further deep dephosphorization, this process produces steelmaking-grade iron, showing potential for industrial application.

## Figures and Tables

**Table 1:** Chemical analysis of the coarse concentrates.

Elements	TFe	SiO_2_	CaO	MgO	Al_2_O_3_	MnO	P_2_O_5_	LOI
Content %	52.65	4.28	1.57	0.14	1.24	0.67	1.95	11.25

**Table 2:** Experimental design.

No.	Coal Blending Ratio/%	Ca(OH)_2_ Dosage/g	Basicity	Reduction Temperature/°C	Melting Separation Temperature/°C	Melting Separation Time/min
1	26	0	0.3	1100	1400	40
2	26	1.5	0.5	1100	1400	40
3	26	5.0	1.0	1100	1400	40
4	26	9.1	1.5	1100	1400	40
5	26	0	0.3	1150	1450	40
6	26	1.5	0.5	1150	1450	40
7	26	5.0	1.0	1150	1450	40
8	26	9.1	1.5	1150	1450	40

**Table 3:** Experimental design.

No.	Coal Blending Ratio/%	Reduction Temperature/°C	Reduction Heating Time/min	Reduction Isothermal Time/min	Melting Separation Time/min
1	30	950	60	120	50
2	30	1000	60	120	50
3	30	1050	60	120	50
4	30	1100	60	120	50

**Table 4:** The optical basicity values of common components in slag.

MgO	CaO	MnO	FeO	Al_2_O_3_	Fe_2_O_3_	SiO_2_	P_2_O_5_
0.8	1.0	0.6	0.48	0.6	0.48	0.48	0.4

## Data Availability

The original contributions presented in this study are included in the article. Further inquiries can be directed to the corresponding author.
